# Facility-based active management of the third stage of labour: assessment of quality in six countries in sub-Saharan Africa

**DOI:** 10.2471/BLT.14.142604

**Published:** 2015-08-31

**Authors:** Linda Bartlett, David Cantor, Pamela Lynam, Gurpreet Kaur, Barbara Rawlins, Jim Ricca, Vandana Tripathi, Heather E Rosen, on behalf of the Quality of Maternal and Newborn Care Study Group of the Maternal and Child Health Integrated Program

**Affiliations:** a Johns Hopkins Bloomberg School of Public Health, 615 North Wolfe Street, Baltimore, MD 21205, United States of America (USA).; b ICF International, Rockville, USA.; c Jhpiego, Nairobi, Kenya.; d Christ Hospital, Cincinnati, USA.; e Jhpiego, Washington, USA.

## Abstract

**Objective:**

To assess the quality of facility-based active management of the third stage of labour in Ethiopia, Kenya, Madagascar, Mozambique, Rwanda and the United Republic of Tanzania.

**Methods:**

Between 2009 and 2012, using a cross-sectional design, 2317 women in 390 health facilities were directly observed during the third stage of labour. Observers recorded the use of uterotonic medicines, controlled cord traction and uterine massage. Facility infrastructure and supplies needed for active management were audited and relevant guidelines reviewed.

**Findings:**

Most (94%; 2173) of the women observed were given oxytocin (2043) or another uterotonic (130). The frequencies of controlled cord traction and uterine massage and the timing of uterotonic administration showed considerable between-country variation. Of the women given a uterotonic, 1640 (76%) received it within three minutes of the birth. Uterotonics and related supplies were generally available onsite. Although all of the study countries had national policies and/or guidelines that supported the active management of the third stage of labour, the presence of guidelines in facilities varied across countries and only 377 (36%) of 1037 investigated providers had received relevant training in the previous three years.

**Conclusion:**

In the study countries, quality and coverage of the active management of the third stage of labour were high. However, to improve active management, there needs to be more research on optimizing the timing of uterotonic administration. Training on the use of new clinical guidelines and implementation research on the best methods to update such training are also needed.

## Résumé

**Objectif:**

Évaluer la qualité de la prise en charge active du troisième stade du travail dans les établissements médicaux en Éthiopie, au Kenya, à Madagascar, au Mozambique, en République-Unie de Tanzanie et au Rwanda.

**Méthodes:**

Entre 2009 et 2012, 2317 femmes hospitalisées dans 390 établissements de santé ont été directement observées, à l'aide d'une analyse transversale, lors du troisième stade du travail. Les observateurs ont constaté l'utilisation de médicaments utérotoniques, de la traction contrôlée du cordon et de massages utérins. Les infrastructures et le matériel nécessaires à une prise en charge active ont été contrôlés et les directives applicables ont été examinées.

**Résultats:**

La plupart des femmes observées (94%; 2173) ont été traitées par ocytocine (2043) ou à l’aide d’un autre utérotonique (130). La fréquence de la traction contrôlée du cordon et des massages utérins ainsi que le moment choisi pour administrer l'utérotonique variaient considérablement d'un pays à l'autre. Parmi les femmes traitées à l’aide d’un médicament utérotonique, 1640 (76%) l’ont reçu dans les trois minutes qui suivent la naissance. Les utérotoniques et le matériel associé étaient généralement disponibles sur place. Si tous les pays étudiés disposaient de politiques et/ou de directives nationales soutenant la prise en charge active du troisième stade du travail, la présence de directives dans les établissements variait selon les pays et seuls 377 (36%) des 1037 prestataires sondés avaient bénéficié d'une formation appropriée au cours des trois années précédentes.

**Conclusion:**

La qualité et le nombre des bénéficiaires de la prise en charge active du troisième stade du travail étaient élevés dans les pays étudiés. Il est cependant nécessaire pour améliorer la prise en charge active de mener davantage de recherches afin d'optimiser le moment d'administration de l'utérotonique. Une formation à l'utilisation de nouvelles directives cliniques et une recherche sur la mise en œuvre des meilleures méthodes pour mettre à jour cette formation sont également nécessaires.

## Resumen

**Objetivo:**

Evaluar la calidad de la gestión activa de la tercera etapa del parto en establecimientos sanitarios de Etiopía, Kenya, Madagascar, Mozambique, Rwanda y la República Unida de Tanzanía.

**Métodos:**

Entre 2009 y 2012, se observaron 2.317 mujeres en 390 establecimientos sanitarios durante la tercera etapa del parto utilizando un diseño transversal. Los observadores registraron el uso de medicamentos uterotónicos, la tracción controlada del cordón y el masaje uterino. Se verificaron las infraestructuras del establecimiento y los suministros necesarios para la gestión activa y se revisaron las directrices relevantes.

**Resultados:**

La mayoría (94%; 2173) de las mujeres observadas recibieron oxitocina (2.043) u otros medicamentos uterotónicos (130). Las frecuencias de la tracción controlada del cordón y el masaje uterino y el momento de la administración uterotónica mostraron considerables diferencias entre países. De las mujeres que recibieron un medicamento uterotónico, 1.640 (76%) lo recibieron dentro de los tres minutos posteriores al nacimiento. Los medicamentos uterotónicos y los suministros relacionados estaban generalmente disponibles en el lugar. A pesar de que los países estudiados tenían políticas nacionales y directrices que apoyaban una gestión activa de la tercera etapa del parto, la presencia de dichas directrices en los establecimientos sanitarios variaba dependiendo del país, y solo 377 (36%) de los 1.037 proveedores investigados habían recibido una formación relevante durante los tres años previos.

**Conclusión:**

En los países estudiados, la calidad y cobertura de la gestión activa durante la tercera etapa del parto eran elevadas. Sin embargo, se necesitan más investigaciones sobre cómo optimizar el momento de la administración uterotónica de cara a mejorar la gestión activa. También hace falta formación en el uso de las nuevas directrices médicas e investigación sobre la aplicación de los mejores métodos para actualizar dichas formaciones.

## ملخص

**الغرض:**

تقييم جودة الإدارة الفعالة للمرحلة الثالثة من المخاض داخل المؤسسات في إثيوبيا، وكينيا، ومدغشقر، وموزامبيق، ورواندا، وجمهورية تنزانيا المتحدة.

**الطريقة:**

تم وضع 2317 امرأة تحت الملاحظة المباشرة بأسلوب مستعرض لعدة قطاعات داخل 390 مؤسسة صحية في الفترة ما بين عامي 2009 و2012، وذلك أثناء مرورهن بالمرحلة الثالثة من المخاض. وقد سجل القائمون على الملاحظة استخدام أدوية مقوية لتوتر الرحم، وسحب الحبل السري المُضبّط، وتدليك منطقة الرحم. وتم فحص البنية التحتية للمنشآت والموارد اللازمة للإدارة الفعالة، كما تمت مراجعة المبادئ التوجيهية المتعلقة بذلك.

**النتائج:**

تم إعطاء معظم النساء اللاتي خضعن للملاحظة (94%؛ 2173) دواء الأوكسيتوسين (2043) أو أي نوع آخر من الأدوية المقوية لتوتر الرحم (130). وأظهر معدل تكرار إجراء سحب الحبل السري المُضبّط، وتدليك منطقة الرحم، وتوقيت إعطاء الأدوية المقوية لتوتر الرحم للنساء، تباينًا فيما بين الدول لا يستهان به. فقد تناولت 1640 (76%) امرأة، من بين النساء اللاتي تم إعطاؤهن الأدوية المقوية لتوتر الرحم، أحد هذه الأدوية في غضون ثلاث دقائق من الولادة. وتوافرت في أغلب الأحيان الأدوية المقوية لتوتر الرحم والمستلزمات ذات الصلة في المواقع المشار إليها. وعلى الرغم من وجود سياسات قومية و/أو مبادئ توجيهية تدعم الإدارة الفعالة للمرحلة الثالثة من المخاض بجميع الدول التي خضعت للدراسة، تباينت نسبة التواجد الفعلي للمبادئ التوجيهية في المنشآت في الدول وتلقى 377 (36%) فقط من مقدمي الخدمة، البالغ عددهم 1037، ممن خضعوا للاستقصاء تدريبًا يتعلق بالخدمة المقدمة في غضون الثلاث سنوات الماضية.

**الاستنتاج:**

ارتفعت نسبة جودة الإدارة الفعالة للمرحلة الثالثة من المخاض واتسع النطاق الذي تقدَّم فيه في الدول التي خضعت للدراسة. وعلى الرغم من ذلك، يتطلب تحسين هذه الإدارة الفعالة إجراء المزيد من الأبحاث حول سبل تحسين توقيت إعطاء الأدوية المقوية لتوتر الرحم للنساء. كما يتطلب الأمر توفير التدريب على استخدام مبادئ توجيهية سريرية جديدة، وإجراء أبحاث حول تنفيذ أفضل الطرق لتعديل هذا النوع من التدريب وفقًا لأحدث المتغيرات

## 摘要

**目的:**

旨在评估埃塞俄比亚、肯尼亚、马达加斯加、莫桑比克、卢旺达以及坦桑尼亚联合共和国的医疗机构中第三产程积极管理的质量。

**方法:**

2009 年至 2012 年间，我们设计了一种横断面调查，对 390 个医疗机构的 2317 名女性在第三产程中进行了直接观察。观察者记录了子宫收缩剂、控制性脐带牵引和子宫按摩的使用。对积极管理的医疗机构基础设施和所需供给进行审核，并且评审了相关指南。

**结果:**

接受观察的大部分女性（94%，2173 名）使用了催产素（2043 名）或另一种子宫收缩剂（130 名）。控制性脐带牵引和子宫按摩的使用频率，以及子宫收缩剂管理的时间选择在各国之间显示出相当大的差异。在使用子宫收缩剂的女性中，有 1640 名 (76%) 是在分娩后 3 分钟内使用。子宫收缩剂和相关产品在生产时使用普遍。尽管所有参与研究的国家都有国家政策和/或指南支持第三产程的积极管理，但是医疗机构现有的指南在各个国家均有差异，受调查的 1037 家供应商中只有 377  (36%) 家供应商在过去 3 年里接受过相关培训。

**结论:**

在参与研究的国家中，第三产程的积极管理质量和覆盖率较高。但是，为了改善积极管理，在优化子宫收缩剂使用时间的选择方面需要进行更多的研究。在新的临床指南方面，还需要对使用者进行培训，并且对最佳方法实施研究，以更新这种培训。

## Резюме

**Цель:**

Оценить качество активного ведения третьего этапа родов в условиях медицинского учреждения в Кении, на Мадагаскаре, в Мозамбике, Объединенной Республике Танзании, Руанде и Эфиопии.

**Методы:**

Мы применили перекрестный метод для анализа работы 390 медицинских учреждений за период с 2009 по 2012 годы. Под наблюдение попали медицинские учреждения, где производилось ведение третьего этапа родов у 2317 женщин. Наблюдатели зафиксировали применение средств, вызывающих сокращение матки, использование контролируемой тракции за пуповину и осуществление массажа матки. Был проведен аудит инфраструктуры медицинских учреждений и расходных материалов, необходимых для активного ведения родов, выполнена проверка соответствующих рекомендаций.

**Результаты:**

Большинству женщин (94%, 2173 роженицы) давали окситоцин (2043 человека) или другое средство для стимуляции сокращения матки (130 человек). Частота выполнения контролируемой тракции за пуповину и массажа матки, а также сроки применения стимуляторов сокращения матки значительно различались в разных странах. Из тех женщин, которым назначался препарат для сокращения матки, 1640 (76%) получали его в течение трех минут после рождения ребенка. Средства для стимуляции сокращений матки и сопутствующие расходные материалы обычно были доступны в медицинском учреждении. Во всех странах, участвовавших в исследовании, существовали национальные правила или рекомендации, которые поддерживают активное ведение третьего этапа родов. Однако применение этих рекомендаций в медицинских учреждениях было различным в разных странах, и только 377 (36%) из 1037 обследованных медицинских работников прошли соответствующее обучение в предшествующие три года.

**Вывод:**

Качество и распространенность активных методов ведения третьего этапа родов в исследованных странах оказались высокими. Однако для улучшения активного ведения родов необходимо провести дополнительные исследования, касающиеся уточнения оптимального времени введения средств, стимулирующих сокращение матки. Также нужны учебные курсы по внедрению новых клинических рекомендаций. Кроме того, практические исследования, посвященные лучшим из таких методов, позволили бы обновить материал подобных учебных курсов.

## Introduction

Haemorrhage is estimated to cause 27.1% of the 287 000 maternal deaths that occur annually.^1^ Postpartum haemorrhage can be prevented by the active management of the third stage of labour – an intervention that can reduce maternal blood loss by up to 66% compared with physiological or expectant management.^1^^–^^3^ While the annual numbers of maternal deaths attributable to haemorrhage fell sharply between 1990 and 2013, postpartum haemorrhage continues to be the global leading cause of maternal death.^4^ The problem does not appear to be a lack of effective interventions but rather the failure to implement such interventions properly in all settings.^1^

Maternal care has traditionally been tracked by two key indicators: the proportion of births attended by skilled birth attendants and antenatal care coverage.^5^ However, these two indicators may not reflect the content or quality of the care available.^6^ For example, the presence of skilled birth attendants does not guarantee that appropriate interventions are correctly implemented at appropriate times. A recent assessment identified 18 quality-of-care indicators for evaluating facility-based deliveries, including the “proportion of women who are administered uterotonics in the third stage of labour.”^7^

Recommendations for specific actions that make up the active management of the third stage of labour have evolved with research. Since 2003, these recommendations have resulted in several attempts to define the essential components of such management (Table 1). In a recent multicentre trial led by the World Health Organization (WHO), it was suggested that use of a uterotonic alone may suffice to prevent postpartum haemorrhage and that “omission of CCT [controlled cord traction] has very little effect on the risk of severe haemorrhage.”^12^ In 2012, based on these findings, WHO issued revised recommendations that emphasized the use of a uterotonic, suggested that controlled cord traction should be optional – and only ever implemented by a skilled birth attendant – and did not recommend the use of sustained uterine massage.^11^ Delayed cord clamping, which appears to benefit the neonate, is also now recommended.^13^^,^^14^

**Table 1:** Components of active management of the third stage of labour in various guidelines

Source of definition	Administration of uterotonic	Timing of uterotonic administration	Controlled cord traction	Uterine massage	Delayed cord clamping
FIGO/ICM (2003)^8^	Recommended	Within a minute of the birth	Recommended	Recommended	Not mentioned
WHO (2007, 2009)^9^^,^^10^	Recommended	Soon after birth	Recommended	Recommended	Recommended
WHO (2012)^11^	Recommended	In third stage of labour	Optional	Optional	Recommended

FIGO: International Federation of Gynaecology and Obstetrics; ICM: International Confederation of Midwives; WHO: World Health Organization.

There have been few reports on the coverage and quality of the active management of the third stage of labour in developing countries. In a global survey it was found that only 16 (43%) of 37 countries investigated included administration of a uterotonic and/or the active management of the third stage of labour in their national health management information systems.^15^ Often, any quality indicators relating to postpartum haemorrhage prevention are monitored non-systematically at subnational level and then only in the context of specific projects. A study done in seven countries in 2005–2006 reported that the active management of the third stage of labour was only implemented correctly in 0.5–32% of the deliveries observed.^16^ No study since has had a similar size and scope and used observation to assess such management.

To provide a baseline for future measurement and inform policy and programme interventions, we assessed the quality and coverage of the active management of the third stage of labour in facility-based deliveries in six countries in sub-Saharan Africa. We investigated the separate components of such management – focusing on uterotonic provision to reflect the most recent research and guidelines. The relevant national policies – if any – and the availability of the various commodities needed for such management were also assessed.

## Methods

### Study design

With a cross-sectional design, we used direct observation of facility-based labour and delivery to assess quality of care in normal delivery practice and the management of selected complications during active management of the third stage of labour. For each of our six study countries, a routine checklist for the clinical observation of labour and delivery (available from the corresponding author) was adapted from a previous study^16^ and partly based on the *Managing complications in pregnancy and childbirth: a guide for midwives and doctors* manual.^17^ There were only minor differences between the six checklists: each was piloted during the training of the data collectors. Lessons from the first two countries where the survey was implemented – i.e. Ethiopia and Kenya – helped refine the tools used elsewhere.

In each study facility, we audited the infrastructure and supplies needed and reviewed whether national policies and/or practice guidelines supported the active management of the third stage of labour. Providers were interviewed and tested on their knowledge of maternity care. In five of our study countries, data were collected, using customized forms, on smartphones or tablet computers. In Kenya, however, data were recorded on paper.

Our data collectors were midwives and doctors who were currently in clinical practice. Clinical refresher training was offered before the collectors were trained as observers. The latter training included four days in a classroom followed by one or two days of pretesting the data collection form – in all the study countries except Kenya – on smartphones or tablets. In role-play simulations based on the MamaNatalie and NeoNatalie models (Laerdal, Stavanger, Norway), trainees assumed the roles of observer, client and health-care provider and practised using the checklists for uncomplicated and complicated births. At the end of the training, data collectors also visited a nearby non-study facility to practise using the checklist in the field.

### Study setting

The data for this study were collected, between 2009 and 2012, in surveys in Ethiopia, Kenya, Madagascar, Mozambique, Rwanda and the United Republic of Tanzania (Table 2). Each survey, which took two to four weeks to complete, was supported by the United States Agency for International Development via the Maternal and Child Health Integrated Program and facilitated by staff at the programme’s headquarters in Washington, United States of America, the programme’s country office in each study country and the six corresponding ministries of health. At the time of survey implementation, the maternal mortality ratio, in deaths per 100 000 live births, ranged from 440 in Madagascar to 790 in the United Republic of Tanzania. In five of our six study countries, approximately 35–55% of women gave birth in facilities and nearly all pregnant women made at least one visit to an antenatal care clinic. Ethiopia had the lowest percentages of facility-based births (10%) and of pregnant women receiving antenatal care at least once (34%).^18^

**Table 2:** Survey samples used to study the active management of the third stage of labour in six countries, sub-Saharan Africa, 2009–2012

Sample	Ethiopia	Kenya	Madagascar	Mozambique	Rwanda	United Republic of Tanzania	Total
**Facilities visited**	19	409	36	46	72	61	643
**Facilities with deliveries**	18	170	36	46	64	56	390
Hospitals	18	150	27	21	42	17	275
Health centres and dispensaries	0	20	9	25	22	39	115
**Deliveries observed**	192	626	347	525	293	706	2689
**Deliveries with third stage of labour observed**	117	564	288	507	225	616	2317

### Participants

Women were approached as they arrived at the labour and delivery ward, received a description of the study by the observer and those that consented to participate were followed. There were up to three women per observer and several observers per facility. If a woman who came in had a complication – such as pre-eclampsia – or if she developed a complication during labour, she would be prioritized for observation.

Overall, 2689 women consented to observation and 2317 of these women were observed during the third stage of labour and therefore included in our final analysis (Table 2). Although 643 health facilities were visited, the number visited in each study country varied widely – from 19 in Ethiopia to 409 in Kenya (Table 2). Only the 390 visited facilities where labour and delivery were observed were included in the final analysis. The other 253 either did not offer labour and delivery services or had no clients during the observation period.

### Study size

All samples, except that of Tanzania, were believed to be nationally representative of facilities with at least moderately high utilization (Table 3).^20^ In Kenya, the survey was implemented within a national Service Provision Assessment run by ICF Macro (Calverton, USA). Ethiopia’s sample was limited to hospitals with at least five deliveries per day. In Madagascar, the sample included all facilities with at least two deliveries daily. Rwanda’s survey was a census of district and referral hospitals and a random selection of district health centres. The two surveys in the United Republic of Tanzania were planned to serve as the baseline and endline of a quality improvement project run by the Maternal and Child Health Integrated Program and only included facilities in project regions.

**Table 3:** Sample framework used to study the active management of the third stage of labour in six countries, sub-Saharan Africa, 2009–2012

Country	Sampling frame	Facility selection	Facility type	Geographical distribution
Ethiopia	2008–2009 AMDD assessment of EmOC	By delivery caseload – all facilities with at least five deliveries per day	Central and specialized, regional, zonal, and district hospitals	Five of the nine regions plus Addis Ababa and Dire Dawa
Kenya	Ministry of health list of facilities	Selected to be nationally representative^19^	National referral, provincial, district, sub-district, and other hospitals, health centres, clinics, dispensaries and maternities	National
Madagascar	2009 UNFPA/AMDD assessment of EmOC	By delivery caseload – all MCHIP-supported facilities with at least two deliveries per day	Regional, district, and teaching hospitals and health centres^a^	17 of the 22 regions
Mozambique	Ministry of health list of facilities	By delivery caseload – all MCHIP-supported facilities with at least two deliveries per day	Central, district, general, provincial, and rural hospitals and rural and urban health centres	National
Rwanda	Ministry of health list of facilities	By level of facility and location – all district-level and higher hospitals plus one randomly selected health centre per district	District, military, and teaching/referral hospitals and health centres	National
United Republic of Tanzania	Facilities that were MAISHA-supported in 2009	By level of facility and delivery caseload – all MAISHA-supported facilities with at least one delivery per day	Regional hospitals, health centres and dispensaries^b^	15 of the 30 regions

AMDD: averting maternal death and disability; EmOC: emergency obstetric and neonatal care; MAISHA: Mothers and Infants, Safe Healthy Alive; MCHIP: Maternal and Child Health Integrated Program; UNFPA: United Nations Population Fund.

^a^ Including three facilities, in three different regions, that did not have at least three deliveries per day.

^b^ Two of the investigated regions had no health centres that had at least one delivery per day. In each of these two regions, the facility with the highest delivery caseload was surveyed.

### Variables

At the time that our study was conceived in 2008, the International Federation of Gynaecology and Obstetrics/International Confederation of Midwives’ definition of the active management of the third stage of labour was still widely used. This definition includes uterotonic administration within a minute of the birth, controlled cord traction and uterine massage.^8^ We collected data on each of these components and also on the components of the relaxed definition^16^ that included uterotonic administration within three minutes of the birth.^9^^,^^21^ The type of uterotonic administered – if any – was recorded. Variables were created based on “yes” or “no” responses to checklist items. Any “do not know” responses were excluded. Analyses of the timing of uterotonic administration were based on observers’ recordings of the times. If not recorded, the timing of administration was assumed to have been more than three minutes after the birth. Kenyan observers estimated the timing of administration as at delivery of the anterior shoulder, within a minute of the baby’s delivery or after placental delivery.

### Statistical analysis

The data for each study country were analysed separately. Post-stratification weights were applied to the observations to account for differences between the numbers of observed and expected deliveries at each facility. Weights were based on the relevant national health management information systems or facility registers. For each study country, descriptive statistics were generated separately for each investigated component of the active management of the third stage of labour and for the combination of all such components.

Facilities were assessed for the presence of at least one non-expired dose of oxytocin, ergometrine or misoprostol that was onsite – i.e. in the delivery room or a neighbouring room. Such drugs were recorded as “not present” if the observer did not personally see a dose.

### Ethical considerations

The study protocol was approved by ethical review boards in each country and by the Johns Hopkins Bloomberg School of Public Health, which ruled that the protocol was exempt from review under the United States Code of Federal Regulations, 45 CFR 46.101(b)(5). Informed consent was obtained from all study participants, including facility directors, health workers and patients.

## Results

Providers with nurse or midwifery training performed most of the observed deliveries in each study country (Table 4). In the knowledge test, 440 (42%) of the 1037 providers investigated indicated that, in the previous three years, they had received pre-service or in-service training in delivery care but only 377 (36%) said that they had received training in the active management of the third stage of labour (Table 5).

**Table 4:** Qualifications of providers observed performing deliveries in six countries, sub-Saharan Africa, 2009–2012

Qualification	No. of providers (%)						
	Ethiopia (*n* = 192)	Kenya (*n* = 626)	Madagascar (*n* = 347)	Mozambique (*n* = 525)	Rwanda (*n* = 293)	United Republic of Tanzania (*n* = 706)	Total (*n* = 2689)
Physician^a^	39 (20)	6 (1)	65 (19)	1 (< 1)	6 (2)	13 (2)	130 (5)
Nurse or midwife^b^	137 (71)	614 (98)	258 (74)	433 (82)	260 (89)	627 (89)	2329 (87)
Non-qualified staff^c^	0 (0)	6 (1)	1 (< 1)	52 (10)	2 (1)	45 (6)	106 (4)
Student^d^	9 (5)	0 (0)	21 (6)	23 (4)	13 (4)	11 (2)	77 (3)
Other or unknown^e^	7 (4)	0 (0)	2 (1)	16 (3)	12 (4)	10 (1)	47 (2)

^a^ General practitioners, obstetricians, gynaecologists, other specialists, resident junior doctors and – in the United Republic of Tanzania – assistant medical officers.

^b^ Bachelor of science, diploma, registered and enrolled nurses, bachelor of science, diploma, registered and enrolled midwives, nurse/midwives and nursing officers. Also includes health officers in Ethiopia, paramedics in Madagascar and maternal and child health aides in the United Republic of Tanzania.

^c^ Medical attendants, health assistants and traditional birth attendants.

^d^ In Mozambique this category included resident junior doctors.

^e^ In Kenya this category included students.

**Table 5:** Self-reported training in previous three years of providers who were observed delivering babies in six countries, sub-Saharan Africa, 2009–2012

Focus of training	No. of providers (%)						
	Ethiopia (*n* = 79)	Kenya (*n* = 234)	Madagascar (*n* = 138)	Mozambique (*n* = 186)	Rwanda (*n* = 145)	United Republic of Tanzania (*n* = 255)	Total (*n* = 1037)
Delivery care	40 (51)	82 (35)	41 (30)	105 (56)	63 (43)	109 (43)	440 (42)
AMTSL	30 (38)	72 (31)	29 (21)	91 (49)	58 (40)	97 (38)	377 (36)

AMTSL: active management of the third stage of labour.

**Fig. 1:**
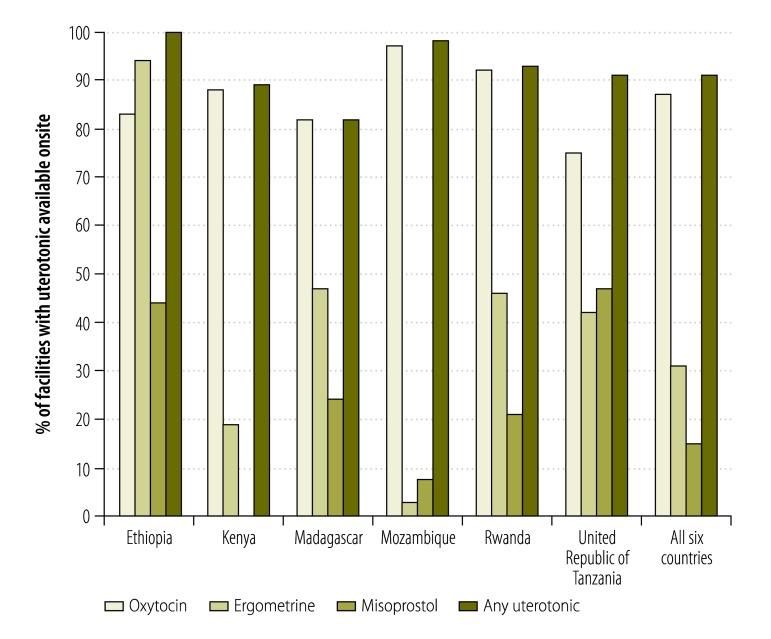
Availability of uterotonics in health facilities in six countries, sub-Saharan Africa, 2009–2012

Note: The plotted data only relate to the 378 facilities – 18 in Ethiopia, 170 in Kenya, 34 in Madagascar, 40 in Mozambique, 61 in Rwanda and 55 in the United Republic of Tanzania – in which data on uterotonic availability were collected.

Data on the availability of a uterotonic in the delivery room were missing for 12 of the 390 facilities included in the final analysis. Of the remaining 378 facilities, 344 (91%) and 329 (87%) had at least one uterotonic and oxytocin available in the delivery room, respectively. Only 41 (75%) of the 55 Tanzanian facilities included in the final analysis had oxytocin available onsite – with more hospitals stocking the drug than health centres (Fig. 1). The syringes and needles needed to administer oxytocin were available in almost all facilities. Availability of ergometrine and misoprostol varied widely. Of the 378 facilities, 166 (44%) – including only four (22%) of the 18 Ethiopian facilities – displayed clinical guidelines for a normal delivery, that included the provision of active management of the third stage of labour, either on a wall or in another easily visible location.

For routine deliveries, each study country included the active management of the third stage of labour – including all components in the International Federation of Gynaecology and Obstetrics/International Confederation of Midwives definition^21^ and oxytocin as the preferred uterotonic – in its service delivery guidelines. In each country’s essential drug list, oxytocin was registered and indicated for use in the active management of the third stage of labour. All of the relevant national policies noted that any provider who was considered to be a skilled birth attendant was eligible to administer uterotonics.

### Individual management components

In the 2317 deliveries observed, uterotonic administration was nearly universal (Table 6). Oxytocin was the most frequently used uterotonic. Among the study countries, Kenya demonstrated the highest frequency of controlled cord traction and uterine massage. Of the 2173 women given a uterotonic at any time, 1640 (76%) received it within three minutes of the birth. However, in only 1124 (52%) of the 2173 women given a uterotonic was it administered within a minute of the birth.

**Table 6:** Implementation of components of the active management of the third stage of labour in six countries, sub-Saharan Africa, 2009–2012

Component	No. of deliveries (%)^a^						
	Ethiopia (*n* = 117)	Kenya (*n* = 564)	Madagascar (*n* = 288)	Mozambique (*n* = 507)	Rwanda (*n* = 225)	United Republic of Tanzania (*n* = 616)	Total (*n* = 2317)
Deliveries any uterotonic given DUG (% of deliveries)	114 (97)	531 (94)	243 (84)	454 (90)	221 (98)	610 (99)	2173 (94)
Oxytocin was given (% of DUG)	112 (98)	522 (98)	242 (100)	453 (100)	220 (100)	494 (81)	2043 (94)
Ergometrine was given (% of DUG)	2 (2)	4 (1)	1 (< 1)	0 (0)	1 (< 1)	25 (4)	33 (2)
Syntometrine was given (% of DUG)	0 (0)	5 (1)	0 (0)	1 (< 1)	0 (0)	1 (< 1)	7 (< 1)
Misoprostol was given (% of DUG)	0 (0)	0 (0)	0 (0)	0 (0)	0 (0)	90 (15)	90 (4)
Uterotonic was given < 1 minute after birth (% of DUG)	90 (79)	422 (79)	99 (41)	156 (34)	55 (25)	302 (50)	1124 (52)
Uterotonic was given 1–3 minutes after the birth (% of DUG)	15 (13)	0 (0)^b^	81 (33)	163 (36)	88 (40)	169 (28)	516 (24)
Uterotonic was given > 3 minutes after the birth (% of DUG)	9 (8)	109 (21)	63 (26)	135 (30)	78 (35)	134 (22)	528 (24)
Controlled cord traction was performed (% of deliveries)	92 (79)	499 (88)	171 (59)	269 (53)	166 (74)	464 (75)	1661 (72)
Uterine massage was performed (% of deliveries)	49 (42)	496 (88)	158 (55)	360 (71)	107 (48)	361 (59)	1531 (66)
Any AMTSL component was performed (% of deliveries)	114 (98)	562 (100)	254 (88)	490 (97)	224 (100)	611 (99)	2255 (97)
AMTSL was performed within 1 minute of birth (% of deliveries)	35 (30)	352 (62)^b^	52 (18)	84 (17)	21 (9)	178 (29)	722 (31)
AMTSL was performed within 3 minutes of birth (% of deliveries)	40 (34)	352 (62)^b^	107 (37)	174 (34)	62 (28)	261 (42)	996 (43)

AMTSL: active management of the third stage of labour.

^a^ Percentages shown represent the values obtained after weighting according to each surveyed facility’s delivery caseload.

^b^ In Kenya, uterotonic administrations within 1 and 3 minutes of the birth were not distinguished.

Note: Percentages have been rounded.

Fifty of the women observed developed postpartum haemorrhage and all but one of these 50 women had been given oxytocin. The other woman had not received any uterotonic.

## Discussion

In all six of our study countries, the quality and coverage of the active management of the third stage of labour were high. The practice of at least one component of such active management was nearly universal. Uterotonic administration was the most frequently observed component and is generally considered to be the most important.^11^ However, there was wide variation among the study countries in the use of controlled cord traction, uterine massage and the timing of uterotonic administration.

Encouragingly, skilled birth attendants conducted almost all of the observed deliveries, uterotonics and other related supplies were usually present onsite and all of the study countries had national policies or guidelines for the active management of the third stage of labour. However, the surveys revealed a low frequency of provider training in active management during the previous three years and the frequent unavailability in delivery rooms of relevant guidelines.

In our study, almost as many women received a uterotonic more than one minute after the birth as within a minute of the birth. Confusingly, there are many differing recommendations on when a uterotonic should be administered. A review of active versus expectant management for women in the third stage of labour, found six recommendations, including “at the delivery of the anterior shoulder”, “immediately following birth” and “within two minutes of birth”.^3^ The International Federation of Gynaecology and Obstetrics/International Confederation of Midwives definition^8^ recommended “within one minute” – whereas the 2007^22^ and 2009^10^ WHO guidelines recommended “soon after birth of the baby”. The most recent – i.e. 2012 – WHO guidelines simply recommended “during the third stage of labour”.^11^ The need for further information on the optimal timing of uterotonic administration has been identified in almost all of the relevant WHO guidelines, trial reports and Cochrane reviews since 2007.^3^^,^^11^^,^^12^^,^^22^^–^^25^ However, neither in a five-country assessment of the impact of all components of the active management of the third stage of labour^26^ nor in an eight-country assessment of such active management with and without controlled cord traction^12^ was the timing of uterotonic administration discussed.

Confusion over changing definitions and guidelines is a barrier to optimal implementation of the active management of the third stage of labour. Studies from Colombia, Ghana and the United Republic of Tanzania have concluded that the lack of uniformity in definitions may contribute to the creation of barriers to effective dissemination of knowledge, consistent training, and implementation of clinical guidelines in practice.^27^^,^^28^^,^^9^Many health facilities in low-resource countries are under-staffed so that a single provider may need to manage several deliveries concurrently and may be unable to provide all of the recommended interventions at the recommended times – even when the necessary supplies are available.^29^^,^^30^ Given the current focus on uterotonic use, future research and guidelines should define the upper and lower time-limits for uterotonic administration to prevent postpartum haemorrhage.

The presence of confusing guidelines, low provision of training and lack of monitoring of content have previously been identified as barriers to optimal implementation of the active management of the third stage of labour.^16^ In 2012, it was observed that the providers of active management need improved educational and training opportunities.^15^ A multifactorial intervention – using clinical leaders, clear service delivery guidelines, regular reviews and supportive materials – could improve the implementation of active management.^31^ The development of appropriate standards and guidelines and clinical audits could promote a so-called culture of quality throughout a country’s health facilities and systems.^32^

The active management of the third stage of labour in Ethiopia and the United Republic of Tanzania has been assessed in 2005–2006.^16^ We also surveyed these two countries in 2010. Comparisons between the data indicate that progress has been made in both countries. However, sampling differences and changing definitions mean that such comparisons have to be handled with care. Since 2005, both countries have developed their first national policies and guidelines for the prevention of postpartum haemorrhage. The percentage of observed Tanzanian women who received a uterotonic within one minute of the birth rose from 10% in 2005–2006 to 50% in 2010 and oxytocin represented 31% and 81% of the uterotonic doses observed in 2005–2006 and 2010, respectively. The percentage of observed Ethiopian women who received a uterotonic within one minute of the birth rose from 41% in 2005–2006 to 79% in 2010. Over the same period, the percentage of oxytocin use increased from 68% to 98%.

The use of direct observation – which remains rare in the assessment of obstetric quality of care – may be considered a strength of this study. However, it also allows potential bias. Observers’ judgments – even if standardized through training and assessed using inter-rater reliability measures – may not be correct. Further, the observer’s presence may have stimulated improvements in the performance of the observed provider.^33^ The surveys were limited to observing care practices for facility-based deliveries only and do not provide data on home births. In a recent study of uterotonic use after delivery that included both facilities and homes, it was estimated that only 40% of Tanzanian women received a uterotonic^34^ – a value much lower than the 99% recorded by us in health facilities. While we used a wide variety of sampling strategies, the surveys were nationally representative and used standardized approaches for the assessment of active management that enabled cross-country comparisons. This study built local capacity to conduct direct observational research and collected baseline data that should be useful in future assessments. Based on these survey tools, a new index has been developed to measure the quality of facility-based labour and delivery care. This should make it quicker and easier to repeat such assessments.^35^

Our analysis focuses primarily on the process component of quality of care – i.e. the actual health care given to patients.^36^^–^^38^ Although we present some information on the human and material resources,^37^^,^^38^ our study was not designed to assess quality of care based on outcomes.^38^ A full evaluation of the quality of the active management of the third stage of labour would require assessment of the inputs, processes, outputs and outcomes.

Although we found evidence of progress being made since 2005, there is still room for improvement. As new evidence becomes available and revisions to global guidelines are developed, national policies and guidelines should also be updated. As an organization responsible for setting global standards in health practice, WHO is in the best position to ensure that new guidelines are introduced in countries. National guidelines, in turn, should stimulate appropriate training and the production of updated standard management guidelines that are readily available at the facility level.^39^ National health management information systems should include uterotonic provision to enable regular local tracking of the quality of active management in the third stage of labour. Implementation research should be done to inform the best ways to introduce and use new guidelines at the facility level.

## References

[1] Say L, Chou D, Gemmill A, Tunçalp Ö, Moller AB, Daniels J, et al. Global causes of maternal death: a WHO systematic analysis. Lancet Glob Health. 2014 6;2(6):e323–33. 10.1016/S2214-109X(14)70227-X 25103301

[2] Khan KS, Wojdyla D, Say L, Gülmezoglu AM, Van Look PF. WHO analysis of causes of maternal death: a systematic review. Lancet. 2006 4 1;367(9516):1066–74. 10.1016/S0140-6736(06)68397-9 16581405

[3] Begley CM, Gyte GM, Devane D, McGuire W, Weeks A. Active versus expectant management for women in the third stage of labour. Cochrane Database Syst Rev. 2011; (11):CD007412. 22071837 10.1002/14651858.CD007412.pub3 PMC4026059

[4] Kassebaum NJ, Bertozzi-Villa A, Coggeshall MS, Shackelford KA, Steiner C, Heuton KR, et al. Global, regional, and national levels and causes of maternal mortality during 1990–2013: a systematic analysis for the Global Burden of Disease Study 2013. Lancet. 2014 9 13;384(9947):980–1004. 10.1016/S0140-6736(14)60696-6 24797575 PMC4255481

[5] Millennium Development Goals indicators [Internet]. New York: United Nations Statistics Division; 2008. Available from: http://unstats.un.org/unsd/mdg/Host.aspx?Content=Indicators/OfficialList.htm [cited 2015 Feb 25].

[6] Hodgins S. Achieving better maternal and newborn outcomes: coherent strategy and pragmatic, tailored implementation. Glob Health Sci Pract. 2013 8;1(2):146–53. 10.9745/GHSP-D-13-00030 25276527 PMC4168574

[7] Bonfill X, Roqué M, Aller MB, Osorio D, Foradada C, Vives A, et al. Development of quality of care indicators from systematic reviews: the case of hospital delivery. Implement Sci. 2013;8(1):42. 10.1186/1748-5908-8-42 23574918 PMC3626798

[8] Joint statement: management of the third stage of labour to prevent post-partum haemorrhage. The Hague: International Confederation of Midwives; 2003. Available from: http://www.pphprevention.org/files/ICM_FIGO_Joint_Statement.pdf [cited 2015 Mar 3]. 10.1016/j.jmwh.2003.11.005 14710151

[9] Mfinanga GS, Kimaro GD, Ngadaya E, Massawe S, Mtandu R, Shayo EH, et al. Health facility-based active management of the third stage of labor: findings from a national survey in Tanzania. Health Res Policy Syst. 2009;7(1):6. 10.1186/1478-4505-7-6 19371418 PMC2676279

[10] WHO guidelines for the management of postpartum haemorrhage and retained placenta. Geneva: World Health Organization; 2009. 23844453

[11] WHO recommendations for the prevention and treatment of postpartum haemorrhage. Geneva: World Health Organization; 2012. Available from: http://apps.who.int/iris/bitstream/10665/75411/1/9789241548502_eng.pdf [cited 2015 Feb 25]. 23586122

[12] Gülmezoglu AM, Lumbiganon P, Landoulsi S, Widmer M, Abdel-Aleem H, Festin M, et al. Active management of the third stage of labour with and without controlled cord traction: a randomised, controlled, non-inferiority trial. Lancet. 2012 5 5;379(9827):1721–7. 10.1016/S0140-6736(12)60206-2 22398174

[13] Leduc D, Senikas V, Lalonde AB, Ballerman C, Biringer A, Delaney M, et al.; Clinical Practice Obstetrics Committee; Society of Obstetricians and Gynaecologists of Canada. Active management of the third stage of labour: prevention and treatment of postpartum hemorrhage. J Obstet Gynaecol Can. 2009 10;31(10):980–93. 19941729 10.1016/S1701-2163(16)34329-8

[14] Rabe H, Reynolds G, Diaz-Rossello J. Early versus delayed umbilical cord clamping in preterm infants. Cochrane Database Syst Rev. 2004; (4):CD003248. 15495045 10.1002/14651858.CD003248.pub2

[15] Smith J, Currie S, Perri J, Bluestone J, Cannon T. National programs for the prevention and management of postpartum hemorrhage and pre-eclampsia/eclampsia: a global survey, 2012. Baltimore: Maternal and Child Health Integrated Program; 2012.

[16] Stanton C, Armbruster D, Knight R, Ariawan I, Gbangbade S, Getachew A, et al. Use of active management of the third stage of labour in seven developing countries. Bull World Health Organ. 2009 3;87(3):207–15. 10.2471/BLT.08.052597 19377717 PMC2654655

[17] Managing complications in pregnancy and childbirth: a guide for midwives and doctors. Geneva: World Health Organization; 2007.

[18] Ethiopia demographic and health survey 2011, preliminary report. Addis Ababa: Central Statistical Agency; 2011. Available from: http://dhsprogram.com/pubs/pdf/PR10/PR10.pdf [cited 2015 Jun 15].

[19] Kenya service provision assessment (SPA) 2010. Nairobi: National Coordinating Agency for Population and Development; 2011. Available from: http://dhsprogram.com/pubs/pdf/SPA17/SPA17.pdf [cited 2015 Jun 22].

[20] Maternal and newborn quality of care surveys [Internet]. Baltimore: Maternal and Child Health Integrated Program; 2013. Available from: http://www.mchip.net/QoCSurveys [cited 2015 Jan 18].

[21] Stanton CK, Deepak NN, Mallapur AA, Katageri GM, Mullany LC, Koski A, et al. Direct observation of uterotonic drug use at public health facility-based deliveries in four districts in India. Int J Gynaecol Obstet. 2014 10;127(1):25–30. 10.1016/j.ijgo.2014.04.014 25026891

[22] WHO recommendations for the prevention of postpartum haemorrhage. Geneva: World Health Organization; 2007.

[23] Gülmezoglu AM, Widmer M, Merialdi M, Qureshi Z, Piaggio G, Elbourne D, et al. Active management of the third stage of labour without controlled cord traction: a randomized non-inferiority controlled trial. Reprod Health. 2009;6(1):2. 10.1186/1742-4755-6-2 19154621 PMC2647525

[24] Soltani H, Hutchon DR, Poulose TA. Timing of prophylactic uterotonics for the third stage of labour after vaginal birth. Cochrane Database Syst Rev. 2010; (8):CD006173. 20687079 10.1002/14651858.CD006173.pub2

[25] Begley CM, Gyte GM, Devane D, McGuire W, Weeks A. Active versus expectant management for women in the third stage of labour. Cochrane Database Syst Rev. 2015;3:CD007412. 25730178 10.1002/14651858.CD007412.pub4

[26] Sheldon WR, Durocher J, Winikoff B, Blum J, Trussell J. How effective are the components of active management of the third stage of labor? BMC Pregnancy Childbirth. 2013;13(1):46. 10.1186/1471-2393-13-46 23433172 PMC3607929

[27] Miranda JE, Rojas-Suarez J, Paternina A, Mendoza R, Bello C, Tolosa JE. The effect of guideline variations on the implementation of active management of the third stage of labor. Int J Gynaecol Obstet. 2013 6;121(3):266–9. 10.1016/j.ijgo.2012.12.016 23528800

[28] Schack SM, Elyas A, Brew G, Pettersson KO. Experiencing challenges when implementing active management of third stage of labor (AMTSL): a qualitative study with midwives in Accra, Ghana. BMC Pregnancy Childbirth. 2014;14(1):193. 10.1186/1471-2393-14-193 24903893 PMC4057904

[29] Haeri S, Dildy GA 3rd. Maternal mortality from hemorrhage. Semin Perinatol. 2012 2;36(1):48–55. 10.1053/j.semperi.2011.09.010 22280866

[30] Nyamtema AS, Urassa DP, van Roosmalen J. Maternal health interventions in resource limited countries: a systematic review of packages, impacts and factors for change. BMC Pregnancy Childbirth. 2011;11(1):30. 10.1186/1471-2393-11-30 21496315 PMC3090370

[31] Althabe F, Bergel E, Cafferata ML, Gibbons L, Ciapponi A, Alemán A, et al. Strategies for improving the quality of health care in maternal and child health in low- and middle-income countries: an overview of systematic reviews. Paediatr Perinat Epidemiol. 2008 1;22(s1) Suppl 1:42–60. 10.1111/j.1365-3016.2007.00912.x 18237352

[32] Raven J, Hofman J, Adegoke A, van den Broek N. Methodology and tools for quality improvement in maternal and newborn health care. Int J Gynaecol Obstet. 2011 7;114(1):4–9. 10.1016/j.ijgo.2011.02.007 21621681

[33] Landsberger HA. Hawthorne revisited: management and the worker, its critics, and developments in human relations in industry. Ithaca: Cornell University; 1958.

[34] Ricca J, Dwivedi V, Varallo J, Singh G, Pallipamula SP, Amade N, et al. Uterotonic use immediately following birth: using a novel methodology to estimate population coverage in four countries. BMC Health Serv Res. 2015;15(1):9. 10.1186/s12913-014-0667-1 25609355 PMC4307135

[35] Tripathi V, Stanton C, Strobino D, Bartlett L. Development and validation of an index to measure the quality of facility-based labor and delivery care processes in sub-Saharan Africa. PLoS ONE. 2015;10(6):e0129491. 10.1371/journal.pone.0129491 26107655 PMC4479466

[36] Donabedian A. The quality of care. How can it be assessed? JAMA. 1988 9 23-30;260(12):1743–8. 10.1001/jama.1988.03410120089033 3045356

[37] Morestin F, Bicaba A, Sermé JD, Fournier P. Evaluating quality of obstetric care in low-resource settings: building on the literature to design tailor-made evaluation instruments–an illustration in Burkina Faso. BMC Health Serv Res. 2010;10(1):20. 10.1186/1472-6963-10-20 20089170 PMC2837005

[38] Campbell SM, Roland MO, Buetow SA. Defining quality of care. Soc Sci Med. 2000 12;51(11):1611–25. 10.1016/S0277-9536(00)00057-5 11072882

[39] Smith JM, Currie S, Cannon T, Armbruster D, Perri J. Are national policies and programs for prevention and management of postpartum hemorrhage and preeclampsia adequate? A key informant survey in 37 countries. Glob Health Sci Pract. 2014 8;2(3):275–84. 10.9745/GHSP-D-14-00034 25276587 PMC4168639

